# Effective hands-on teaching strategy on participants’ confidence in medical writing and publishing: A before-after study

**DOI:** 10.1371/journal.pone.0307681

**Published:** 2024-07-26

**Authors:** Behrooz Astaneh, Hadi Raeisi Shahraki, Vala Astaneh, Gordon Guyatt

**Affiliations:** 1 Faculty of Health Sciences, Department of Health Research Methods, Evidence and Impact, McMaster University, Hamilton, Ontario, Canada; 2 Department of Epidemiology and Biostatistics, School of Health, Shahrekord University of Medical Sciences, Shahrekord, Iran; 3 Faculty of Kinesiology and Health Sciences, York University, Toronto, Ontario, Canada; 4 Department of Health Research Methods, Evidence and Impact and Department of Medicine, McMaster University, Hamilton, Ontario, Canada; Iran University of Medical Sciences, ISLAMIC REPUBLIC OF IRAN

## Abstract

Proficiency in medical writing is crucial for disseminating reports of medical studies. The impact of workshops in this regard on participants’ confidence is a subject of debate. We assessed the impact of a hands-on workshop on participants’ confidence in medical writing. Participants of a 2-day “learning-by-doing” workshop held at McMaster University participated in this before-after study. We used a unique, reliable, and valid tool comprising two domains of confidence in medical writing and using English language before and after receiving the educational intervention. Of 25 participants, 21 completed the instrument before and after the workshop. Typical participants were female, and students in their 30s, who had not attended a prior workshop. The mean (95% CI) increase in the participants’ confidence for domain 1 was 15.3 (10.5, 20.1), for domain 2 was 16.8 (9.8, 23.8), and for the total score was 32.1 (20.9, 43.2) (all P<0.001). Between-subgroup analyses showed the score increase was significantly higher in participants with less than 5 years of experience in medical research. The workshop had a positive impact on enhancing participants’ confidence in writing skills, including using active verbs, crafting short sentences, summarizing main findings, and adhering to checklists like CONSORT. Hands-on medical writing workshops can boost participants’ confidence in writing medical articles and using optimal English language. Targeting junior researchers and graduate students could result in a better outcome. Emphasizing the writing areas where participants achieved higher score changes might yield better outcomes for such workshops.

## Introduction

Proficiency in medical writing and a thorough understanding of international standards for medical publishing are crucial for disseminating reports of medical studies [1,2]. Publishing is the last stage of research, without which the whole process of conception, design, data gathering, analyses, and interpretations would be largely wasted [3]. Within the academic landscape of health sciences, attaining success in medical publishing not only establishes personal credibility but also serves as a pivotal criterion for academic promotion [4].

Acquiring knowledge about elements associated with medical writing and publishing, including the standard structure of medical articles [5], ethical issues related to publishing, including authorship criteria [6,7], knowing the recommended guidelines and required items that should be considered in different types of articles [8,9], how to deal with medical journals, and how to select a journal [10], are essential for medical researchers to feel confident in medical writing [11].

Currently, the English language is the lingua franca of academic medical publishing [12]. For non-native English users, writing medical articles in English presents a considerable challenge, contributing to barriers in publishing and diminished confidence compared to native users [13–15]. Cultural inaccuracies, manuscript structure, and issues with grammar and style present notable challenges [16]. Non-native English users often exhibit common patterns such as varied usage of prepositions and an overreliance on passive verbs in their writing [17].

While essential elements of medical writing and publishing, along with optimal English skills, could be incorporated into graduate health sciences programs, the absence of a formal course dedicated to medical writing and publishing in research-focused medical education curricula necessitates the provision of such skills through specialized workshops [18].

Confidence refers to “people’s sense of competence and skill, their perceived capability to deal effectively with various situations” [19]. So, in medical writing, confidence means feeling competent and skillful in writing a standard medical article. The impact of these workshops on participants’ confidence remains a subject of debate. Debate persists due first to variations in teaching strategies and secondly to the lack of a standardized measurement tool with approved validity and reliability to assess confidence in dealing with the challenges of medical writing and publishing. Consequently, our objective was to assess the impact of a hands-on medical writing and publishing workshop on participants’ confidence in medical writing and the use of optimal English for this purpose using a unique, reliable and valid measurement tool.

## Methods

### Study design

This was a before-after study.

### Study population

Participants of a 2-day workshop on medical writing and publishing held on 8–9 Nov 2023 at McMaster University, Hamilton, Ontario, Canada, were eligible to participate in the study. The target audience for our workshop was clinical researchers in the early or middle stages of their careers. Students in graduate programs, clinical researchers, post-doctoral fellows, and junior and mid-career faculty members were among the primary target group, although other participants, such as professionals from industry who felt the workshop could be of benefit to them, would also be welcome.

### Delivery of the workshop

The 2-day workshop marked a pioneering initiative at McMaster University, led by five faculty members from the Health Research Methods, Evidence, and Impact (HEI) Department, alongside three editors from the Canadian Medical Association Journal (CMAJ). These facilitators brought extensive expertise in medical writing and publication, with two of them having a track record of successfully leading similar workshops in the past.

The mode of delivery of the workshop was “learning-by-doing,” in which the participants had the opportunity to offer a draft of a manuscript at hand at the time of participation for discussion and feedback. During the workshop and in the related breakout rooms, we anticipated that discussion and feedback conducted under the supervision of facilitators and using the workshop content would improve these articles, ideally to be ready for submission to a reputable peer-reviewed journal.

The workshop curriculum aimed to comprehensively cover the fundamentals and essential aspects of manuscript development, encompassing various topics related to standards and ethical publication (Table 1). Sessions addressed the standard structure of medical articles, including the Introduction, Methods, Results, and Discussion (IMRaD) sections. Additionally, the workshop addressed aspects of publication ethics, authorship criteria, insights into medical journal operations, and guidelines for reporting different article types (e.g., CONSORT, STROBE, PRISMA). Preceptors offered practical advice on responding to peer reviewers, understanding scholarly metrics, and selecting journals. The workshop dedicated specific attention to the optimal use of English in medical article writing, illustrating key points with examples from published articles.

**Table 1:** Detailed agenda of the 2-day hands-on medical writing/publishing workshop held at McMaster University in November 2023.

Topic	Timeline	Session-specific learning objectives
**DAY 1 (08. Nov.2023)**		
Participant Introductions	8:30–8:50	1- Introduce participants to one another
Topic	Timeline	Session-specific learning objectives
Expectations and summarising your message	8:50–9:20	1. Expectations of the workshop 2. Summarising the message of your study in one sentence
How to Write an Introduction	9:20–9:50	1- identify the importance of writing Introduction by moving from general ideas to the focused research question 2- outline the Problem-Gap-Hook heuristic as another approach to writing an introduction
Break	9:50–10:05	
So You Think You Know Your English? Common mistakes that even the most fluent English-speaking authors make	10:05–11:05	1- Suggestions for optimizing language use and flow of ideas throughout the manuscript
Interactive Writing Session #1 – Writing the Introduction	11:05–12:50	Facilitators will be joining groups to optimize discussion. The groups will be encouraged to review manuscripts to consider strengths and limitations and how to improve
Lunch & Networking,	12:05–12:55	
How to Write a Methods Section	12:55–13:35	1- The importance and basic elements for writing the method 2- Special considerations in writing the method of RCTs 3- Special considerations in writing the method of SR/MAs
How do Medical Journals Operate?	1:35–2:35	Insights on: 1- medical journals workflow, 2- common pitfalls of submissions 3- when to appeal a decision 4- what will (on average) increase the likelihood of success
Break	2:35–2:50	
Getting to know different reporting guidelines (CONSORT, PRISMA, STROBE, STARD, RECORD….)	2:50–3:20	Insight on: 1- How guidelines can help writing a paper 2- Different guidelines for different studies
Interactive Writing Session #2 Writing the Method section	3:20–4:20	Facilitators will be joining groups to optimize discussion. The groups will be encouraged to review manuscripts to consider strengths and limitations and how to improve
Mentorship	4:20–4:50	1- discuss the importance of having a good mentor and how he/she can help junior researchers during their academic career
**DAY 2 (09. Nov.2023)**		
How to Write Your Results Section	8:30–9:15	1- describe the importance of a results section, and how to organise it 2- compare and contrast different parts of a results section including “text”, “Tables”, and “Figures” and will learn how to implement them in their articles. 3-Special considerations in writing the method of RCTs 4-Special considerations in writing the method of SR/MAs
Interactive Writing Session #3 Writing the Results section	9:15–10:15	Facilitators will be joining groups to optimize discussion. The groups will be encouraged to review manuscripts to consider strengths and limitations and how to improve
Break	10:15–11:00	
Authorship Criteria, Ghost Authorship, Guest Authorship	10:30–11:00	1- understand different debates about authorship in medical articles 2- describe the ICMJE standard requirements for biomedical authorship 3- contrast authorship, contributorship, and other alternative ways of providing acknowledgement to members of your team
How to Write Your Discussion Section	11:00–11:30	Optimal structure and content of the Discussion
Responding to Peer Reviewers	11:30–12:00	1- identify the tips on how to respond to reviewers rapidly and efficiently 2- Editors view on responding to reviewers
Lunch & Networking,	12:00–1:00	
Journal Selection & Journal Metrics	1:00–1:30	1- describe how to select the most suitable journal for their article in different databases and across different publishers and where not to publish 2- list different metrics used wrongly rank journals and researchers (IF, H index, …)
Interactive Writing Session #4 Writing the Discussion	1:30–2:30	Facilitators will be joining groups to optimize discussion. The groups will be encouraged to review manuscripts to consider strengths and limitations and how to improve
Break	2:30:2:45	
Writing the Title & Abstract	2:45–3:15	1- list the importance of Titles and different types of Titles in medical articles
Interactive Writing Session #5 Writing the Title and Abstract	3:15–4:00	Facilitators will be joining groups to optimize discussion. The groups will be encouraged to review manuscripts to consider strengths and limitations and how to improve
Publication Ethics	4:00–4:15	1- Discuss the importance 2- Ignored cases (redundant publication, salami publication, … 3- COPE flowcharts
Wrap Up & Reflections	4:15–4:30	Reflect on the last two days

### Measurements

We informed the participants of the workshop about the aims of the study and asked them to participate. We included those who signed written informed consent forms. We gathered data about participants’ demographic characteristics and administered an instrument evaluating their confidence in their medical writing and publishing skills. After the workshops, we assessed the participants’ confidence again with the same tool.

### Measurement instrument

We had previously created a measuring tool by creating an item pool after a comprehensive literature search. After rating and ranking the items by a team of professionals, a tool was created whose psychometric properties were checked in a sample of previous workshop applicants by using standard statistical analysis [20]. The instrument has 36 items comprising two domains. Domain 1 is “confidence in choosing the appropriate contents for different parts of a standard medical article based on the international guidelines” (18 items), and domain 2 is “confidence in the use of appropriate academic English language required for publishing articles in peer-reviewed medical journals” (18 items). We prepared a Google form with the response options with a 5-point Likert scale ranging from 1 (showing no confidence) to 5 (extremely confident) (S1 File). So, the range of scores in each domain was from 18 to 90. Higher scores show higher confidence.

The total content validity index of the tool was 0.75. The internal consistency of the tool was greater than 0.90 for both domains. Temporal stability was checked using test-retest reliability with r = 0.92.

### Sample size

The sample size was determined first by feasibility issues, including the institutional resources to hold the workshop and ultimately by the number who proved interested and enrolled.

### Outcome measures

The primary outcome was considered to be an increase in confidence scores in both domains and in total after the educational intervention. The secondary outcome was an increase in the score of individual items after the intervention. An increase in mean scores greater than 1 for individual items was considered a more pronounced impact of the educational intervention.

### Ethics board approval

This study was reviewed by the Hamilton Integrated Research Ethics Board (HIREB) and approved with the code 2023-14916-GRA.

### Analysis of data

We reported demographic variables descriptively using mean±SD or proportion as required. For between-group analysis, we used the independent *t* test and one-way analysis of variance (ANOVA) followed by the LSD post hoc test. Analyses addressed the pre-post changes in the confidence scores in both domains of the measurement instrument using a paired *t* test. The software for statistical analysis was SPSS version 23.

## Results

In total, 31 applicants registered for the workshop. Two applicants changed their decision to participate because of schedule conflicts. We sent an email to the remaining 29 applicants, informing them of the aims and procedure of the before-after study, and asked for their consent to participate in the study; 25 consented. We emailed the Google form containing the measurement tool addressing their confidence to them 2 days before the workshop and asked them to fill out the form prior to the start of the workshop. We sent two reminder emails to those who had not filled out the forms before the workshop.

Of the 25 participants, 21 completed the instrument before starting the workshop. Two had forgotten to fill out the form before the first day, and the remaining two filled out the form after starting the workshop on day one, so we excluded them from the study.

After the workshop, the same form was emailed to the 21 participants who were included in the study. All returned the filled form within a few days of receipt.

The mean (± SD) age of the participants in the study was 35.4 (± 8.6) years (range: 17–52). 14 (66.7%) were female, and 14 (66.7%) were students. Two (9.5%) were faculty members, and nine (42.9%) had doctoral education. Typical participants were students in their 30s, who had not attended a prior similar workshop. Table 2 shows the participants’ demographic characteristics and the number of publications as the first author and workshops attended.

**Table 2:** Demographic variables, publications, and workshop attendance of participants in a before-and-after study performed during a 2-day hands-on workshop on medical writing and publishing held at McMaster University.

Variable	Subgroup	Frequency	Percent
Sex	Female	14	66.7
	Male	7	33.3
Education	BSc	2	9.5
	MSc	7	33.3
	Doctorate	9	42.9
	Postdoc	1	4.8
Academic career	Student	14	66.7
	Faculty member	2	9.5
	staff	5	23.8
Number of previous workshops attended	None	13	61.9
	1–3	4	19.0
	4–6	0	0
	7–9	0	0
	10 or more	2	9.5
Number of publications as the first author	None	3	14.3
	1–3	9	42.9
	4–9	3	14.3
	10 or more	4	19.0
Number of years working in medical research	< 5 years	11	52.4
	6–10 years	6	28.6
	More than 10 years	2	9.5

We encountered a few instances of missing data from two participants, resulting in the sum of frequencies for some variables not rounding to 21.

Table 3 shows that mean pre-test scores in both domains and total scores of the measurement tool increased substantially after the workshop.

**Table 3:** Comparison of pre-test scores with the post-test scores in both domains and in total on the confidence in writing different parts of a standard medical article and using optimal English language in participants of the medical writing workshop held at McMaster University.

Variable	Pre-test	Post-test	Mean difference (95% CI)	P-value
Domain 1	58.3±12.0	73.6± 10.0	15.3 (10.5, 20.1)	<0.001
Domain 2	51.3± 16.4	68.1± 12.4	16.8 (9.8, 23.8)	<0.001
Total score	109.7± 26.6	141.8± 21.2	32.1 (20.9, 43.2)	<0.001

To find possible differences between different subgroups, we did between subgroups analyses, which showed that the mean score changes were statistically significant exclusively among the three groups with different years of experience in medical research (P = 0.04 for domain 1 and = 0.03 for domain 2). We then did the post hoc analysis, which revealed that the score increase was significantly higher in participants with less than 5 years of experience compared with the participants with 6–10 years of working in medical research for both domains (Table 4). Table 4 also shows score changes within different subgroups.

**Table 4:** The comparison of confidence score increase among different subgroups of participants of the medical writing workshop held at McMaster University.

Variable	Subgroup (No)	Domain 1	Within subgroup comparison P-value	Between subgroups P-value	Domain 2	Within subgroup comparison P-value	Between subgroups P-value
		Mean increase (95% CI)			Mean increase (95% CI)		
Sex	Female (14)	15.5 (10.1, 20.9)	<0.001	0.90	16.6 (9.0, 24.3)	<0.001	0.95
	Male (7)	14.9 (2.2, 27.4)	0.03		17.1 (-1.5, 35.8)	0.07	
Education	BSc (2)	23.0 (10.3, 35.7)	0.03	0.74	21.5 (-23.0, 66.0)	0.10	0.96
	MSc (7)	16.7 (6.9, 26.6)	0.01		15.4 (-1.3, 32.1)	0.06	
	Doctorate (9)	14.6 (5.3, 23.8)	0.01		19.0 (5.5, 32.5)	0.01	
	Post doc (1)	10.0	‐‐‐		14.0	‐‐‐	
Academic career	Student (14)	17.6 (10.7, 24.4)	<0.001	0.32	22.0 (13.2, 30.8)	<0.001	0.07
	Faculty member (2)	6.5 (-50.7, 63.7)	0.39		12.5 (6.1, 18.8)	0.03	
	Staff (5)	12.4 (6.1, 18.6)	0.01		4.0 (-9.8, 17.8)	0.47	
Number of prior workshops	None (13)	18.0 (10.7, 25.3)	<0.001	0.47	17.9 (6.8, 29.0)	0.004	0.99
	1–3 (4)	10.5 (4.6, 16.4)	0.01		16.8 (-2.8, 36.3)	0.07	
	4–6 (0)	‐‐‐	‐‐‐		‐‐‐	‐‐‐	
	7–9 (0)	‐‐‐	‐‐‐		‐‐‐	‐‐‐	
	≥10 (2)	14.0 (-36.8, 64.8)	0.18		18.0 (-32.8, 68.8)	0.14	
Number of publications	None (3)	18.3 (9.6, 27.0)	0.01	0.69	11.7 (-21.9, 45.2)	0.27	0.43
	1–3 (9)	16.9 (8.1, 25.6)	0.002		20.1 (8.6, 31.6)	0.004	
	4–9 (3)	9.0 (2.4, 15.6)	0.03		6.7 (-33.8, 47.1)	0.55	
	≥10 (4)	17.5 (7.6, 42.6)	0.11		25.0 (-5.5, 55.6)	0.08	
Number of years working in medical research (years)	≤ 5 (11)	20.8 (13.3, 28.3)	<0.001	**0.04**	25.5 (14.9, 36.0)	<0.001	**0.03**
	6–10 (6)	8.7 (3.6, 12.7)	0.01		5.1 (-4.5, 14.8)	0.23	
	≥10 (2)	13.0 (-12.4, 38.4)	0.10		12.5 (6.1, 18.9)	0.02	

**Fig 1:**
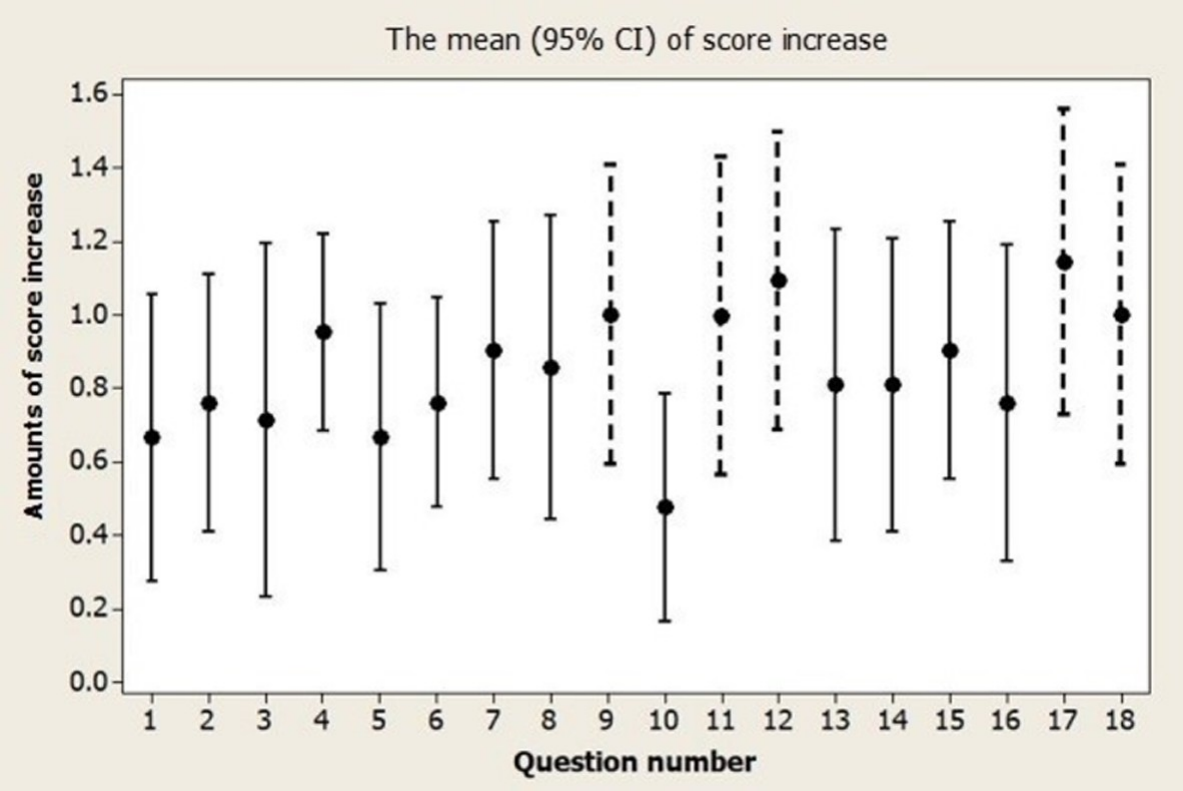
The scores increased for each question of domain 1, with dashed lines indicating questions whose mean increases exceeded 1 unit on the Likert scale.

**Fig 2:**
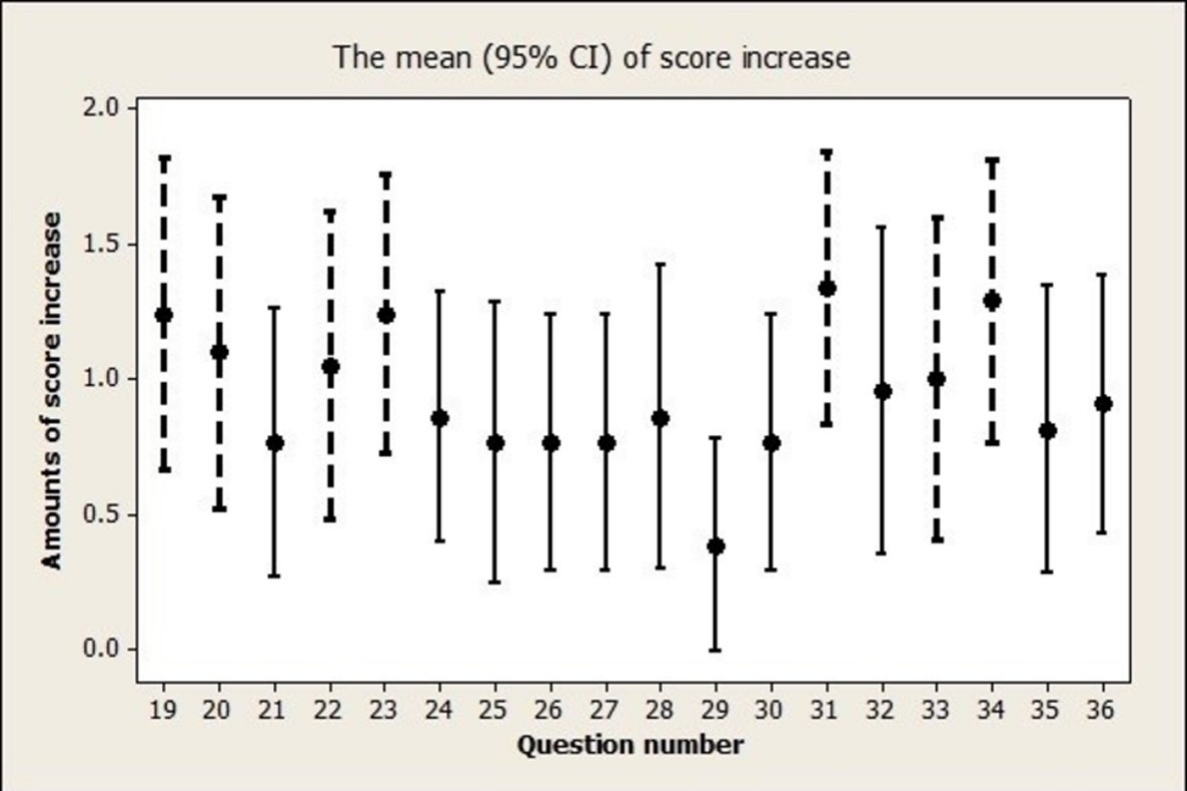
The scores increased for each question of domain 2, with dashed lines indicating questions whose mean increases exceeded 1 unit on the Likert scale.

Figs 1 and 2 show the score increase for each question in both domains. The workshop had a positive impact on enhancing participants’ confidence in various writing skills. This included utilizing figures in the Results section (item 9 in Fig 1), composing the Acknowledgment section (item 11 in Fig 1), summarizing the main findings (item 12 in Fig 1), crafting the conclusion (item 17 in Fig 1), and adhering to checklists such as CONSORT (item 18 in Fig 1). Additionally, participants reported increased confidence in effectively using active verbs (item 19 in Fig 2), crafting short sentences (item 22 in Fig 2), employing simple words (item 23 in Fig 2), incorporating subordinate clauses (item 34 in Fig 2), utilizing fewer words (item 31 in Fig 2), and distinguishing between while and although (item 33 in Fig 2).

## Discussion

We found that the 2-day hands-on workshop at McMaster University effectively enhanced participants’ confidence in writing various sections of a standard medical article and using optimal English language for this purpose. Post-test total scores as well as post-test scores in both domains, substantially increased after the workshop. We found no significant between-subgroups differences except for the participants with varying years of experience in working in medical research. Notably, the mean score changes were significantly more pronounced in participants with less than 5 years of experience compared with those with 6–10 years of experience in medical research for both domains. The reason for this difference could be that those who worked less than five years in the research field had a lower base of knowledge and experience in writing a standard medical article. Therefore, when they received the educational intervention, their scores increased more significantly compared to those with more years of experience in research. As a result, the difference between pre- and post-intervention scores was statistically significant. The non-significant difference in other groups might be related to the lower number of participants in those groups, as there were only 6 participants with 6–10 years of experience.

### Strengths and limitations

This workshop on medical writing and publishing marks a significant milestone as the first to employ a standard, valid, and reliable measurement tool for evaluating participants’ confidence in writing medical articles and using optimal English language.

Another notable strength of this study is the detailed reporting of confidence score changes for each individual question. This level of granularity in reporting, which is unique to our study, has not been observed in similar research endeavours in this field.

The composition of our diverse facilitation team is another strength of this study. Our team included different stakeholders, including world-class researchers, editors from one of the most reputable Canadian medical journals, and facilitators with extensive experience in conducting numerous workshops of this nature.

The diversity of participants, encompassing students from various fields of study, individuals with diverse academic careers, and participants with varying numbers of previous publications, attendance at similar workshops, and years of experience working in medical research is another strength. This diversity can add to the generalizability of the findings.

However, this study has its limitation, notably the small sample size. This is a common constraint in face-to-face, hands-on workshops, primarily due to budget limitations and logistical challenges. In certain subgroups, there were only two participants, which may account for statistically non-significant findings, potentially reflecting the limited statistical power rather than an actual absence of an effect.

### Relation to prior work

In a comparable workshop designed for post-doctoral fellows, the program spanned three weeks and included three 6-hour modules. Facilitators focused on teaching participants techniques for writing with clear and cohesive language. Confidence self-assessments were conducted before and after the workshop, revealing an increase in confidence levels from 1.12 to 1.44 points on a 5-point Likert scale [21]. However, unlike our study, the authors did not furnish details about the content of the confidence tool, and it remained unclear whether the researchers evaluated the psychometric properties of the measurement tool.

In another study aimed at enhancing writing skills for publications, the authors employed remote teleconferencing to teach writing standards to Australian employees within the public hospital system. Utilizing a self-reported 5-point Likert scale questionnaire, with a response rate of 26%, participants noted an increase in their confidence in facing challenges related to writing for publication. However, in contrast to our methodology, the study did not utilize a valid and reliable tool to assess confidence, and there was no report on the content of the questionnaire [22].

Wajekar and colleagues conducted a medical writing workshop for post-graduate anesthesia students, focusing on teaching them how to write a case report. They reported using a validated questionnaire to assess participants’ confidence levels in academic writing skills post-workshop and noted a significant improvement in participants’ confidence levels after the workshop. However, a notable limitation is that they neither reported on the psychometric properties of the questionnaire nor cited a reference for the tool used [23].

A similar limitation is evident in the report of a protocol writing workshop conducted for general practitioners, where pre- and post-workshop questionnaires were administered to participants to gauge their improvement in writing protocols. Participants reported a significant increase in their confidence in writing a research protocol on a 6-point Likert scale. However, the authors did not provide an explanation of how they ensured the validity of the scale used and how the scores could be translated to an actual increase in confidence [24].

In a recent report, Harvey and colleagues detailed their series of three 90-minute face-to-face workshops conducted over an 8-month period for employees of public sector health services, focusing on how to write for publication [25]. Their before-and-after program surveys, comprising both quantitative and qualitative questions, indicated a 0.3 increase in the average confidence level in the ability to write a manuscript for publication on their 5-point Likert scale [25]. Even considering the difference between the number of questions in their questionnaire and our measurement tool, the mean increase in confidence in both domains of our tool in our study was significantly higher than theirs.

Other researchers planning to conduct educational interventions for academic writing and publishing have reported a common challenge: the absence of systematic or validated tools for evaluating such educational courses [15,26]. Sabouni and colleagues reported their intervention aimed at enhancing the level of knowledge and confidence in academic writing and publishing for Syrian participants in 2021. They mentioned using questions from a previous similar course with permission from the copyright holders but did not provide a citation to the previous work [26]. Regarding the impact of their workshop, they stated that the number of participants reporting confidence in writing an article increased from 55% to 83%. Despite acknowledging English as the preferred language for scientific articles, which could pose a challenge for non-native English language users, including their participants, the researchers neither focused on teaching optimal English language nor assessed the participants’ abilities in using optimal English language [26]. An important distinction in our study is the emphasis on teaching optimal English language use and evaluating how this instruction could enhance our participants’ confidence in utilizing the English language for medical writing.

In general, previous reports on similar workshops exhibit inconsistencies in providing comprehensive details about the educational activities, presentation methods, and curricula, often lacking completeness and varying widely across different workshops. Assessing the impact of such workshops on participants’ confidence in academic medical writing has frequently been limited by the absence of rigorous, standardized methods. Valid and reliable measurement tools for these assessments have been notably scarce. Our study distinguishes itself by employing a standard, valid, and reliable measurement tool. We further contribute by reporting mean score changes for individual questions, offering valuable insights for the planning of future workshops by researchers. Additionally, the sharing of the entire measurement tool through this article adds to the distinctiveness of our approach compared to previous reports.

### Implications for practice and research

We presented the mean confidence score changes for individual questions, highlighting items that exhibited an increase in mean scores greater than 1. Such items may signify areas where the workshop could have a more pronounced impact. Notably, participants derived greater benefit from topics covering the appropriate use of figures in the Results section, summarizing main findings, and writing conclusions, as well as utilizing checklists like CONSORT, compared to other items on the measurement tool. Therefore, for future workshops, facilitators can, in addition to maintaining a focus on teaching these subjects, explore improved techniques such as providing some published articles and comparing the good examples with less satisfactory papers for teaching other items that our workshop did not demonstrate a clear impact. This approach aims to enhance the effectiveness of the workshop across a broader range of topics.

We observed that participants with less than 5 years of experience exhibited significantly greater mean score changes in both domains compared to other groups with different years of experience. So, these workshops might be more advisable to actively target junior researchers and graduate students for a more impactful outcome.

## Conclusion

This workshop provided an opportunity to generate evidence related to optimizing the educational strategies of similar workshops and contributed to the literature on their evaluation. Hands-on medical writing and publishing workshops have the potential to boost participants’ confidence in writing various sections of a standard medical article while also improving their proficiency in using the optimal English language for this purpose. Specifically, targeting junior researchers and graduate students could result in a better outcome in participants’ writing abilities. Developing a pre-workshop curriculum emphasizing the writing areas where participants achieved higher score changes might yield better outcomes for such workshops.

## Supporting information

S1 File Final tool for confidence in medical writing. (PDF)

## References

[1] Cargill M, O’Connor P. Writing Scientific Research Articles: Strategy and Steps. Hoboken, New Jersey: Wiley-Blackwell; 2013.

[2] Cahn PS, Benjamin EJ. Academic Writing Workshop for Medical School Faculty. MedEdPORTAL. 2012;8:9289. 10.15766/mep_2374-8265.9289.

[3] Cabanas G, Bridgeman MB, Hermes-DeSantis ER. Publish or perish: Success with publication in pharmacy residency training. Curr Pharm Teach Learn. 2018;10(12):1647–51. doi: 10.1016/j.cptl.2018.08.017 30527833

[4] May May Y, Shih-Hui L, Anshul K, Anne WT. Evaluation of the promotion criteria in an academic medical centre in Singapore. BMJ Lead. 2023:leader-2023-000881. 10.1136/leader-2023-000881. PMC12038089 37890988

[5] Astaneh B, Masoumi S. Professional medical writing and ethical issues: a developing country’s perspective. European Science Editing. 2011;37(3):85.

[6] Astaneh B, Schwartz L, Guyatt G. Biomedical Authorship: Common Misconducts and Possible Scenarios for Disputes. J Acad Ethics. 2021;19(4):455–64. 10.1007/s10805-021-09435-z.

[7] Barbour V, Astaneh B, Irfan M. Challenges in publication ethics. Ann R Coll Surg Engl. 2016;98(4):241–3. doi: 10.1308/rcsann.2016.0104 26985812 PMC5226037

[8] Shaghaghian S, Astaneh B. Adherence to the Strengthening the Reporting of Observational Studies in Epidemiology Statement in Observational Studies Published in Iranian Medical Journals. Iran J Public Health. 2020;49(8):1520–9. doi: 10.18502/ijph.v49i8.3896 33083329 PMC7554393

[9] Sarveravan P, Astaneh B, Shokrpour N. Adherence to the CONSORT Statement in the Reporting of Randomized Controlled Trials on Pharmacological Interventions Published in Iranian Medical Journals. Iran J Med Sci. 2017;42(6):532–43. doi: 10.1371/journal.pmed.1000326 29184261 PMC5684374

[10] Suiter AM, Sarli CC. Selecting a Journal for Publication: Criteria to Consider. Mo Med. 2019;116(6):461–5. doi: 10.12688/f1000research.15256.2 31911720 PMC6913840

[11] Coverdale JH, Roberts LW, Balon R, Beresin EV. Writing for academia: getting your research into print: AMEE Guide No. 74. Med Teach. 2013;35(2):e926–34. doi: 10.3109/0142159X.2012.742494 23228107

[12] Kamadjeu R. English: the lingua franca of scientific research. Lancet Glob Health. 2019;7(9):e1174. doi: 10.1016/S2214-109X(19)30258-X 31401999

[13] Fox CH, Meijer F. Teaching medical English to foreign-language doctors. Med Educ. 1980;14(5):316–9. doi: 10.1111/j.1365-2923.1980.tb02373.x 7432214

[14] Li Y, Cargill M, Flowerdew J. Teaching English for Research Publication Purposes to Chinese Science Students in China: A Case Study of an Experienced Teacher in the Classroom. J Engl Acad Purp. 2018;35:116–29. 10.1016/j.jeap.2018.07.006.

[15] Heseltine E. Teaching scientific writing to non-native English speakers. Medical Writing. 2013;22(1):13–6. 10.1179/204748012X13560931063591.

[16] Pagel W, Kendall FE, Gibbs HR, editors. Self-Identified Publishing Needs of Nonnative English-Speaking Faculty and Fellows at an Academic Medical Institution; 2002.

[17] Ferrari R. Medical writing for non-native English speakers: burden and opportunity. Medical writing. 2015;Medical writing.

[18] Rathore FA, Mansoor SN. How to conduct a workshop on medical writing: Tips, advice and experience sharing. J Pak Med Assoc. 2015;65(6):665–8. 26060168

[19] Shrauger JS, Schohn M. Self-confidence in college students: Conceptualization, measurement, and behavioral implications. Assessment. 1995;2(3):255–78. 10.1177/1073191195002003006.

[20] Astaneh B, Raeisi Shahraki H, Astaneh V, Guyatt G. Assessment of confidence in medical writing: Development and validation of the first trustworthy measurement tool. PLOS ONE. 2024;19(4):e0302299. doi: 10.1371/journal.pone.0302299 38635566 PMC11025726

[21] Cameron C, Deming SP, Notzon B, Cantor SB, Broglio KR, Pagel W. Scientific Writing Training for Academic Physicians of Diverse Language Backgrounds. Acad Med. 2009;84(4):505–10. doi: 10.1097/ACM.0b013e31819a7e6d 19318790

[22] Duncanson K, Webster EL, Schmidt DD. Impact of a remotely delivered, writing for publication program on publication outcomes of novice researchers. Rural Remote Health. 2018;18(2):4468. doi: 10.22605/RRH4468 29793344

[23] Wajekar AS, Salgaonkar SV, Chincholi IH, Shetty AN. Impact of basic medical writing workshop on case report writing by post-graduate anaesthesia trainees: A pilot study. Indian J Anaesth. 2018;62(7):502–8. doi: 10.4103/ija.IJA_98_18 30078852 PMC6053887

[24] Sheikh A, Levy ML. Writing research protocols: An innovative approach. Asthma Gen Pract. 1999;7(3):39–42. 10.1038/pcrj.1999.22.

[25] Harvey D, Barker R, Tynan E. Writing a manuscript for publication: An action research study with allied health practitioners. Focus on Health Professional Education. 2020;21(2):1–16. 10.11157/fohpe.v21i2.397.

[26] Sabouni A, Chaar A, Bdaiwi Y, Masrani A, Abolaban H, Alahdab F, et al. An online academic writing and publishing skills course: Help Syrians find their voice. Avicenna J Med. 2017;7(3):103–9. doi: 10.4103/ajm.AJM_204_16 28791242 PMC5525463

